# Racial disparities in patients with amputation in an acute care setting in the immediate postoperative period

**DOI:** 10.1002/pmrj.13400

**Published:** 2025-06-05

**Authors:** Antonio Mondríguez‐González, Rachel Xie, Rachel Esparza, Manasi Sheth, Mark Huang

**Affiliations:** ^1^ Shirley Ryan Ability Lab Chicago Illinois USA; ^2^ Department of Physical Medicine and Rehabilitation Northwestern University Feinberg School of Medicine Chicago Illinois USA; ^3^ Midwestern University School of Osteopathic Medicine Glendale Arizona USA; ^4^ Present address: 351 Evergreen Circle Glendale Heights Illinois USA

## Abstract

**Background:**

Racial disparities are present in the U.S. medical system and lead to detrimental health outcomes and reduced quality of life for many patients. These can be seen in the increased number of amputations among patients from underrepresented minority groups, in addition to differences in access to appropriate rehabilitation care in many debilitating diagnoses.

**Objective:**

To examine racial differences in access to rehabilitation consultation and discharge to an inpatient rehabilitation facility (IRF) among patients in an acute care setting following an amputation.

**Methods:**

Retrospective convenience sample at a tertiary care hospital from a limited database. The study included 640 participants ≥18 years of age who had undergone an amputation at an academic hospital between January 2020 and March 2023. Patients <18, pregnant women, prisoners, those who identified as Native Hawaiian due their low number (*n* = 1), or those who had undergone solely phalangeal amputations were excluded. The primary outcomes of this study were the discharge destination after acute care hospital and acute care length of stay.

**Results:**

There was a statistically significant association between age and discharge disposition, with a 2% increase in the likelihood of discharge to IRF for each additional year. The length of stay between different racial and ethnic groups showed statistically significant differences, with Asian patients having the longest (23.3 days) and those identifying as “two or more races” having the shortest (11.9 days) stays. When comparing between White, Black, and Hispanic patients, Black patients had the longest length of stay (19.9 days). Lastly, patients who received physiatry consultation were 20 times more likely to be discharged to IRF.

**Conclusion:**

Patients from underrepresented minority groups had a longer acute care length of stay. Race and ethnicity did not appear to affect the amputation level or discharge disposition, including discharge to acute inpatient rehabilitation. Age and the presence of physiatric consultation had the greatest impact on determining the discharge disposition of patients with amputations.

## INTRODUCTION

Racial and ethnic disparities are a result of many structural and systemic barriers that lead to apparent differences in social, economic, and educational attainment. These inequities often permeate multiple aspects of a person's life and lead to detriments to their overall health and well‐being, otherwise known as social determinants of health. Black or African American and Hispanic or Latino populations have a higher prevalence of multiple chronic diseases such as diabetes^1^ and peripheral artery disease (PAD),^2^ among others, with more complications that include amputation and overall worse clinical outcomes. Although 13% of the general adult population has diabetes, disproportionately high rates of diabetes are found among individuals who identify as American Indian or Alaska Native (14.7%), Hispanic or Latino (12.5%), and Black or African American (11.7%). In comparison, lower rates are seen among individuals who identify as Asian (9.2%) and White (7.5%).^3^ Furthermore, Black or African American individuals are more likely to have diabetes, hypertension, and limb‐threatening ischemia and be either self‐insured or enrolled in Medicaid.^4^

Amputations are becoming increasingly more common. According to the Amputee Coalition, 185,000 amputations occur in the United States every year, with the main etiologies being vascular diseases, such as diabetes and PAD, trauma, and, less commonly, cancer.^5^, ^6^ It has been found that Black or African American individuals are four times more likely to undergo a lower extremity amputation than White individuals.^7^ Additionally, a study showed that the incidence of diabetic foot ulcers (DFUs) and amputations as a result of poorly controlled diabetes was greater in Black or African American and Hispanic or Latino populations when compared to White individuals.^8^ Black or African American individuals were almost twice as likely to undergo an amputation in the first year after a DFU in comparison to White individuals.^9^ Additionally, Black or African American patients are more likely to have complications from PAD and less likely to undergo revascularization and limb‐salvage techniques in an attempt to avoid amputation.^10^, ^11^ It has also been estimated that the 1‐year post‐lower‐extremity‐amputation mortality rate is between 10%–50%, and the 5‐year rate for the same population is between 30%–80%.^12^, ^13^, ^14^, ^15^

Patients who undergo amputation are often discharged to an inpatient rehabilitation facility (IRF) for functional preprosthetic training. There is evidence of racial disparities in discharge disposition for other diagnoses. A study documented that Hispanic patients with burns were less likely to be discharged to IRF than White and Black or African American patients.^16^ A similar study also showed that compared to White survivors of traumatic brain injury (TBI), Black or African‐American and Hispanic or Latino patients with TBI were less likely to be discharged to IRF regardless of medical insurance status.^17^ To our knowledge, no prior studies have examined the presence of disparities in the discharge destination of patients with amputation to an IRF.

The purpose of this study is to further examine racial differences experienced by patients in an acute care setting following amputation in relation to access to rehabilitation consultation and discharge disposition to an IRF. Given the ample amount of evidence of disparities in the process of amputation, the chronic conditions that lead to this outcome, and the process of discharging to IRF in other patient populations, we hypothesize that Black or African American and Hispanic or Latino patients with amputations will have a longer acute care length of stay (LOS), be less likely to be discharged to an IRF and less likely to receive a physiatric consultation than White patients.

## METHODS

### Study participants

Study approval was granted by our university's Institutional Review Board. This was a retrospective convenience sample of the patients from an institutional Enterprise Data Warehouse (EDW), which included patients treated by the surgical department at an urban academic acute care hospital. Furthermore, participants included in this study were ≥18 years of age and had undergone an amputation between January 2020 and March 2023. Patients who were <18, pregnant, prisoners, or those who had undergone solely phalangeal amputations were excluded from the study.

### Study outcomes

The primary outcome of this retrospective study was discharge to IRF after undergoing an amputation. Givenpreviously documented disparities in care among under represented minority groups, we were concerned they would be less likely to be discharged to IRF. The secondary outcome was the LOS.

*Data Collection*. Data were collected retrospectively for patients who underwent amputations from January 2020 to March 2023 through our institution's EDW as a limited data set. The data collected included age, gender, race, ethnicity, medical insurance, type of amputation, hospital LOS (in days), and discharge disposition. Age was grouped in the following categories: 18–24 years of age, 25–34 years, 35–44 years, 45–54 years, 55–64 years and >65 years. The patient's race and ethnicity were grouped according to the post‐1997 Office of Management and Budget Standard and the U.S. Census Bureau.^18^, ^19^ Additionally, we combined race and Hispanic ethnicity to create the following demographic categories: White, Black or African American, Hispanic or Latino, Asian, Native Hawaiian or Pacific Islander, American Indian or Alaska Native (AIAN), and two or more races. Those who declined to respond to their racial or ethnic background were put in a separate category labeled as “declined to respond.” To be categorized as White, the patient had to identify as White and answer “no” to the question regarding Hispanic ethnicity. A similar approach was used for Black and African American participants. Using the definition of the U.S. Census Bureau, we categorized patients as Asian if they identified with the following origins of the Far East, Southeast Asia, or Indian subcontinent: Asian Indian, Chinese, Filipino, Korean, Japanese, Vietnamese, and Other Asian. Due to the small number of Native Hawaiians in our sample (*n* = 1) they were excluded from our analysis.

Concerning amputations, the type or level of amputation was determined by collecting the Current Procedural Terminology (CPT) codes used during that determined admission. Through the CPT codes collected, we divided amputation categories into the following: forequarter, shoulder disarticulation, transhumeral, transradial, wrist disarticulation, transmetacarpal, metacarpal, phalanx, hindquarter, hip disarticulation, transfemoral, knee disarticulation, transtibial, ankle disarticulation, through ankle, midtarsal, transmetatarsal, metatarsal, metatarsophalangeal, and interphalangeal. A detailed breakdown of the CPT codes used can be seen in Table 1. Additionally, information regarding the etiology/risk factors of amputation was also collected and consisted of the following: diabetes, PAD, traumatic, cancer, sepsis, disseminated intravascular coagulation, and osteomyelitis. A detailed breakdown of the different etiologies can be seen in Table 2. Finally, discharge disposition consisted of IRF, acute care, home, skilled nursing facility (SNF), expired, or against medical advice (AMA). The “acute care” category consisted of discharges to an acute care hospital, inpatient hospice, long‐term acute care hospital, and Veterans Affairs Health System facility. The “home” category also consisted of discharges to home with self‐care, home with home health care, home with outpatient services, and shelter.

**TABLE 1:** CPT codes.

Type of amputation	CPT codes
Forequarter	23900
Shoulder disarticulation	23920, 23921
Transhumeral	24900, 24920, 24925, 24930, 24931
Transradial	25900, 25905, 25907, 25909
Wrist disarticulation	25920, 25922, 25924
Transmetacarpal	25927, 25929, 25931
Metacarpal	26910
Phalangeal	26951, 26952
Hindquarter	27290
Hip disarticulation	27295
Transfemoral	27590, 27591, 27592, 27594, 27596
Knee disarticulation	27598
Transtibial	27880, 27881, 27882, 27884, 27886
Ankle disarticulation	27889
Through ankle	27888
Midtarsal	28800
Transmetatarsal	28805
Metatarsal	28810
Metatarsophalangeal	28820
Interphalangeal	28825

Abbreviation: CPT, Current Procedural Terminology.

**TABLE 2:** Amputation etiologies.

Etiologies	No.	Estimate	SE	*p* value
Necrotizing fasciitis	23 (3.6%)	0.495	0.446	.266
Peripheral artery disease	398 (62.2%)	0.379	0.209	.07*
Disseminated intravascular coagulation	2 (0.3%)	15.378	601.883	.979
Detachment	206 (32.2%)	0.028	0.219	.897
Traumatic amputation	125 (19.5%)	0.127	0.228	.578
Sepsis	98 (15.3%)	−0.089	0.236	.704
Cancer	18 (2.8%)	−0.623	0.566	.271
Osteomyelitis	303 (47.3%)	−0.272	0.206	.186
Diabetes	309 (48.3%)	−0.03	0.202	.879

* Indicates slightly significant.

### Statistical analysis

Descriptive statistics were used for the continuous variables such as LOS to determine the central tendency and dispersion of the variables. For multiple categorical variables, cross‐tabulation was performed to assess the relationship between the categories of race and ethnicity and the number of physical medicine and rehabilitation (PM&R) consult orders, number of physical therapy evaluations, gender, and their discharge disposition. A Pearson's chi‐square test was performed to determine where there was a statistical significance for such tables of cross‐tabulation. Logistic regression was performed to confirm if there was an association between the presence of discharge to IRF and other variables. Odds ratios (ORs) for the significant variables that were adjusted for confounding factors were computed in order to determine the ratio of likelihood of discharge to IRF.

After conducting our retrospective chart review, a total of 1290 patients underwent an amputation from January 2020 to March 2023. After excluding patients who underwent solely phalangeal amputations, Native Hawaiians (*n* = 1), and incarcerated individuals (*n* = 1), our study sample comprised 640 patients. A power analysis was made for our study, revealing a power of 1. Additionally, the effect size of our study was 0.04, with an accuracy of 0.59.

## RESULTS

### Demographic data

Detailed information on the patients' demographic data is summarized in Table 3. The gender breakdown of our study consisted of 66.6% male and 33.4% female. Regarding the race and ethnicity of our study sample, 52.0% were White, 25.9% were Black or African American, 14.7% were Hispanic or Latino, 2.3% were Asian, 2.7% were two or more races, and 0.5% were AIAN. Additionally, 1.9% declined to respond to questions regarding race and ethnicity. The ages of the patients were as follows: 46.7% were >65 years of age, 29.1% were between the ages of 55 and 64, 13.4% were between ages 45 and 54, 6.4% were between ages 35 and 44, 2.7% were between ages 25 and 34, and 1.7% were <25 years. There was a statistically significant association between age and discharge disposition (*p* value: .002). Indeed, using predictive statistics, we determined that for each additional year, there was a 2% increase in the likelihood of discharging to an IRF (OR, 1.02 [95% CI, 1.01–1.03]). Regarding the patients' gender, we found no statistically significant association between gender and discharge disposition (*p* value: .55). Lastly, there was no statistically significant association between race/ethnicity and type of amputation (*p* value: .193).

**TABLE 3:** Demographic information.

Demographic variable	No.	Proportion
Age (years)		
65 or older	299	0.467
55–64	186	0.291
45–54	86	0.134
35–44	41	0.064
25–34	17	0.027
18–24	11	0.017
Total	640	1
Race or ethnicity		
American Indian or Alaskan Native	3	0.005
Asian	15	0.023
Declined to respond	12	0.019
Hispanic or Latino	94	0.147
Two or more races	17	0.027
Black or African‐American	166	0.259
White	333	0.520
Total	640	1
Gender		
Male	426	0.666
Female	214	0.334
Total	640	1

### Consult order

Of the participants in our study, 95.1% had a PM&R consult order placed. There was no significant association between race or ethnicity and the placement of the PM&R consult order, as evidenced by the use of Pearson's chi‐square (chi‐square value = 8.5972, df = 6, *p* value = .198) and Fisher's exact test (*p* value = .134). Further analysis was made, and through the use of predictive statistics, it was determined that patients who had a PM&R consult order placed were 20 times more likely to be discharged to IRF when compared to those who were not evaluated by the consult team (OR, 20.1 [95% CI: 4.16–199]).

### Discharge disposition

The information regarding discharge disposition is summarized in Table 4. From January 2020 to March 2023, 40.4% of Black or African American, 54.3% of Hispanic or Latino, and 44.1% of White patients with amputations were discharged to IRF. Subsequently, 6.0% of Black or African American, 3.2% of Hispanic or Latino, and 4.5% of White patients with amputations were discharged to acute care. Furthermore, 24.7% of Black or African American, 9.6% of Hispanic or Latino, and 18.9% of White patients were discharged to SNF. Lastly, 24.1% of Black or African American, 28.7% of Hispanic or Latino, and 30.0% of White patients with amputations were discharged home. Despite these trends, there was no statistically significant association between race or ethnicity and discharge disposition (χ^2^ = 77.425, df = 78, *p* = .497) using a Pearson χ^2^ test.

**TABLE 4:** Patient discharge disposition.

	Patient discharge disposition						
Race or ethnicity	Inpatient rehabilitation facility	Acute care services	Skilled nursing facility	Home	Expired	Against medical advice	Total
Patient discharge disposition							
White	147	15	63	100	5	3	333
Black or African American	67	10	41	40	8	0	166
Hispanic or Latino	51	3	9	27	3	1	94
Asian	4	1	3	7	0	0	15
American Indian/Alaskan Native	1	0	0	2	0	0	3
Two or more races	5	0	2	9	1	0	17
Declined to respond	5	1	3	3	0	0	12
Total	280	30	121	188	17	4	640

### Length of stay

The information on LOS is summarized in Table 5. Of the different racial and ethnic groups studied, Asian patients had the highest LOS of 23.3 days (SD ± 37.4). In contrast, those patients identifying as the two or more races category had the lowest LOS, with approximately 11.9 days (SD ± 12.2). When comparing the LOSs for White, Black or African American, and Hispanic or Latino patients, the LOSs were 12.2 (SD ± 12.2), 19.9 (SD ± 22.7), and 17.5 days (SD ± 16.1), respectively. Furthermore, the differences in the mean lengths of stay of the various race and ethnicity groups were significant (*p* value < .001).

**TABLE 5:** Patient length of stay.

Race or ethnicity	Mean	No.	SD
White	12.2	333	12.2
Black or African American	19.9	166	22.7
Hispanic or Latino	17.5	94	16.1
Asian	23.34	15	37.4
American Indian/Alaskan Native	12.34	3	12.1
Two or more races	11.94	17	12.2
Declined to respond	14.5	12	10.0
Total	15.2	640	17.3

### Amputation etiology

Data analysis demonstrated the breakdown of amputation etiology of the included patients as described in Table 2. Logistic regression was performed to assess whether there was an association between amputation and diagnosis. The results were not statistically significant and were as follows: disseminated intravascular coagulation (15.378, SD ± 601.883, *p* = .979), necrotizing fasciitis (0.495, SD ± 0.446, *p* = .226), PAD (0.379, SD ± 0.209, *p* = .07), traumatic amputation (0.127, SD ± 0.228, *p* = .578), detachment (0.028, SD ± 0.219, *p* = .897), diabetes (−0.03, SD ± 0.202, *p* = .879), sepsis (−0.089, SD ± 0.236, *p* = .704), osteomyelitis (−0.272, SD ± 0.206, *p* = .186), and cancer (−0.623, SD ± 0.566, *p* = .271). PAD was trending toward significance (.379, SD ± 0.209, *p* = .07).

### Amputation levels

A detailed breakdown of the patients' amputation levels is described in Table 6. A total of 752 amputations were performed on the patients in our study. Ninety‐seven percent of our study population underwent lower extremity amputations, and 4.2% underwent upper extremity amputations. Furthermore, 14.8% underwent more than one amputation. Of those who underwent multiple amputations, 93.6% underwent a combination of only lower extremity amputations, 5.3% had a mix of upper and lower extremity amputations, and 1.1% had a combination of only upper extremity amputations performed. Of the total amputations, 95.4% consisted of lower extremity amputations. Transtibial amputations were the most common and accounted for 43.5% of lower extremity amputations, whereas transfemoral and transmetatarsal amputations accounted for 21.4% and 18.8% of lower extremity amputations, respectively. Regarding upper extremity amputations, 35.3% and 32.4% consisted of transradial and transhumeral amputations, respectively. When conducting statistical analysis via Pearson chi‐square, there was no significant association between race or ethnicity and amputation level (χ^2^ = 476.67, df = 558, *p* = .994).

**TABLE 6:** Amputations by race or ethnicity.

Type of amputation	White	Black or African American	Hispanic or Latino	Asian	Two or more races	AIAN	Declined to respond	Total	%
Forequarter	0	1	0	0	0	0	0	1	0.1
Shoulder disarticulation	1	0	0	0	0	0	0	1	0.1
Transhumeral	5	3	2	0	0	0	1	11	1.4
Transradial	7	2	2	0	1	0	0	12	1.5
Wrist disarticulation	1	0	0	0	0	0	0	1	0.1
Transmetacarpal	3	0	1	0	0	0	0	4	0.5
Metacarpal	1	0	0	0	0	0	0	1	0.1
Phalangeal	2	1	0	0	0	0	0	3	0.4
Hindquarter	0	0	1	1	0	0	0	2	0.3
Hip disarticulation	5	1	1	0	0	0	2	9	1.2
Transfemoral	87	44	16	1	4	1	1	154	20.4
Knee disarticulation	19	15	4	1	0	0	1	40	5.3
Transtibial	169	62	57	8	10	2	5	313	42.0
Ankle disarticulation	8	2	2	1	0	0	0	13	1.7
Through ankle	1	1	1	0	0	0	0	3	0.4
Midtarsal	1	2	1	0	0	0	0	4	0.5
Transmetatarsal	58	51	18	4	2	0	2	135	18.0
Metatarsal	9	11	5	1	0	0	1	27	3.6
Metatarsophalangeal	6	1	2	0	0	0	1	10	1.3
Interphalangeal	3	4	0	0	0	0	1	8	1.1
	386	201	113	17	17	3	15	752	100

Abbreviation: AIAN, American Indian or Alaska Native.

## DISCUSSION

Our results revealed that patients from underrepresented minority groups such as Black or African American, Hispanic or Latino, and Asian patients had a significantly longer LOS when compared to White individuals following their amputation at an acute care hospital. Multiple factors could be contributing to these discrepancies in LOS. Some of these factors include varying severity of the amputations, socioeconomic status, lifestyle, access to care, or lower‐quality care experienced by minorities stemming from systemic biases in the U.S. health care system. These findings are similar to an analysis done by Ghosh and colleagues, which revealed that despite White patients being older, having a greater degree of chronic conditions, and having a higher inpatient mortality risk than Black and Hispanic patients, Black and Hispanic patients still demonstrated a significantly longer LOS than White patients.^20^ Furthermore, in this same study, analysis revealed that wealthier patients in the highest socioeconomic quartile had a significantly shorter LOS when compared to patients in the lowest socioeconomic quartile.^20^

In addition to the reasons previously mentioned, the LOS for postamputation patients is likely to be influenced by their home support system, medical insurance plan, access to local therapy and care, and even their determination to make progress in their recovery. Wood and colleagues emphasized the importance of a strong support system and highlighted that married individuals were typically healthier than those unmarried because their spouses would encourage healthier behavior.^21^ Additionally, the influence of medical insurance on patients' LOS cannot be understated, and its impact was further demonstrated by Hughes and colleagues, who found that patients with lower median household income, Medicaid insurance coverage, or uninsured status were more likely to undergo amputation and less likely to undergo limb‐saving revascularization, which subsequently increased their likelihood of requiring rehabilitation therapy during their hospital stay.^22^ On the other hand, a more recent study by Frisullo and colleagues comparing acute stroke outcomes of Western European White and non‐Western European White patients, which included Hispanic, Black, and Asian individuals in Italy, found no significant racial or ethnic differences in LOS, but did note significant racial and ethnic disparities in access to revascularization.^23^ Despite the different health care setting present in Italy when compared to our own, it is still important to note the differences and disparities present in access to care and how they influence patient outcomes. The influence of access to care in the amputee population is highlighted by an analysis performed on a 2011–2017 National Inpatient Sample Data, where one million PAD hospitalizations were examined to document the PAD burden in Hispanic patients and comparing their risk profiles, access to care, and outcomes to those of White patients. They found that Hispanic patients were more likely to present directly to the emergency department, have longer LOS, and have a minor or major amputation while also being less likely to undergo surgical or endovascular revascularization.^24^

Despite these findings, there was no significant association between race and ethnicity with discharge disposition. Our results were consistent with a study by Dillingham and colleagues, where an analysis of 348 patients with dysvascular amputations from 18 hospitals in Maryland and Wisconsin found that the patient's race and ethnicity did not significantly influence their discharge destination.^25^ Yet, there are also findings entailing different rehabilitation diagnoses that conflict with our results. For example, in one study by Vadlamani and colleagues, after assessing for racial disparities in discharge disposition of traumatic brain injury patients, they determined that “Blacks or African Americans” were more likely to be discharged to an IRF instead of home when compared to their White counterparts.^26^ Furthermore, another study analyzing the discharge disposition of hip fracture patients from a Texas trauma registry found that Hispanic or Latino and Black or African American patients were more likely to be discharged home regardless of age. Additionally, they discovered that the presence of specific comorbidities such as chronic kidney disease, type 2 diabetes, alcohol use disorder, and usage of anticoagulants increased patients' probability of being discharged to rehabilitation.^27^ Upon further analysis, our results did reveal that among all of the etiologies, PAD and diabetes were the largest contributors to amputations. These findings are consistent with prior studies, as noted in a review conducted by Beckman et al., which found that major lower extremity amputation occurrences were directly proportional to PAD and diabetes‐induced infection rates.^28^ Although the different amputation etiologies showed no statistically significant association to race and ethnicity, PAD did show near significance, a finding that is unsurprising given the preponderance for PAD complications in underrepresented minority groups.^10^, ^11^ Despite our results conflicting with some existing studies, many reasons could explain this. Given that underrepresented minorities are more likely to have longer LOSs and more complicated hospital courses, this would lead to an increased likelihood of being discharged to an IRF as a result of medical necessity. For instance, Black or African American individuals were 2.5 times more likely to undergo amputations than limb‐salvage procedures^29^ and more likely to have a higher level amputation than White patients, which in turn would lead to a more extensive surgery and more complicated hospital course.^30^ A similar trend has been documented with Hispanic or Latino patients, where they are up to twice as likely to undergo a lower extremity amputation than White patients^31^ and more likely to experience operative complications.^32^ Although discharge disposition was not statistically significant with respect to race and ethnicity, there was a trend in which fewer Black or African American and Hispanics or Latino patients were discharged home when compared to White patients, possibly from having a more complicated hospital course and as a result, requiring more intense rehabilitation and care after their surgery. Though not statistically significant, a noticeably higher number of Black or African American patients were discharged to SNF when compared to White and Hispanic or Latino patients. This trend is consistent with a prior study of a large regional dataset of patients undergoing elective total hip arthroplasties (THA), where Black or African American patients were more likely to be discharged to IRF and SNF when compared to White patients for post‐THA care, in addition to having a significantly higher 90‐day readmission rate and 90‐day mortality likelihood.^33^ Acute care hospital complications may play a significant role in the increased likelihood of admission of patients from underrepresented minorities to a postacute care facility for further care.

Most (95.9%) of patients included in this study had a physiatric consult order placed, with no significant association between the consult order and race or ethnicity. It was noted that patients with a physiatrist evaluation at this particular institution were 20 times more likely to be discharged to an IRF after their acute care hospitalization. Not only is the physiatric consultation an important factor for patients with amputations to discharge to IRF, but it can also be important for decision‐making before amputation, preoperative counseling, and postoperative care of patients with amputations. The physiatrist can help the patient understand the medical and surgical process of amputation, in addition to providing invaluable information in elucidating what life may look like living with an amputation. This can include discussing wound healing, residual limb shaping, phantom pain and sensation, and the eventual prosthetic fitting process. They may also be able to connect patients with limb loss support groups and therapists experienced in amputee rehabilitation and help ensure that patients have access to adequate equipment to promote a safe discharge to the community with maximal independence and function. Yet, it is important to emphasize that this study is limited in its generalizability due to the high use of physiatrist consultation at this institution among all patients with limb loss. Many acute care institutions nationwide may not have easy access to physiatric consultation, and those that do may not have access to a standalone IRF. These barriers to access to PM&R consultants or providers may further be exacerbated in low‐resource communities where individuals from minority groups may be overrepresented.^34^ Therefore, this could lead to disproportionately lower access to PM&R consultants for patients with amputation from underrepresented minority backgrounds and lower socioeconomic statuses and potentially admission to an IRF when indicated. Given the high correlation with an IRF discharge destination, a physiatric consultation may alleviate disparities in race and ethnicity for the care of patients with amputation.

## LIMITATIONS

There were multiple limitations to this study. Despite the power of our study being appropriate, our effect size was significantly small, which decreases the practical applications of our study. This can be resolved by expanding the size of our study sample to encompass multiple sites or retrospectively analyzing more patients. Another important limitation consists of the geographical location of our hospital. Given that it resides in a predominantly urban area, this may limit the generalizability to the population at large. Furthermore, our study did not divide patients by their amputation level for most components of data analysis, limiting analysis of the effects of amputation level on LOS and discharge disposition. Additionally, our study entailed grouping the data of upper extremity amputations with lower extremity amputations and distal amputations with more proximal amputations together to adequately power the study. Furthermore, given that we used a limited data set, there was a limited amount of variables that were available for analysis. As such, we were unable to ascertain disease severity and complexity, surgical complications during their acute hospital stay, and other important socioeconomic variables such as zip code, income, employment, and marital status, which could influence patient outcomes. Due to the nature of our study and how the data was obtained, it was common for patients to have undergone more than one amputation during their acute hospital stay, and we were unable to determine the laterality of their amputations. This, in turn, affected the uniformity of our data. Moreover, it is important to note that most patients in our study had a physiatry consult, likely as a result of this institution's robust PM&R consultation service, a factor that may lead to a lack of generalizability for other acute care hospitals with limited access to physiatric consultation. For the minority of patients that did not receive a consultation, it is possible that these patients were likely deemed to be poor rehabilitation candidates. It was difficult to ascertain the specific reasoning behind the placement or nonplacement of PM&R consult orders. This study did not look at patients' functional status and their candidacy for rehabilitation, which could affect their discharge disposition and LOS. Future opportunities to strengthen this study include further analysis of the important socioeconomic factors mentioned here in order to more accurately and holistically highlight their impact in this patient population. Lastly, collaborating with various acute care hospitals from different geographical regions with different levels of access to PM&R consults and IRFs should be pursued in future studies to determine if geographical and/or hospital differences exist and significantly influence outcomes in this patient population.

## CONCLUSION

Our study has found that the LOS in the acute care hospital differs based on race, with patients who identify as Black or African American, Hispanic or Latino, Asian, and Native Hawaiians or Pacific Islander having longer LOSs. Race and ethnicity did not appear to affect the amputation level or discharge disposition, including discharge to acute inpatient rehabilitation. The two factors that appeared to have the most impact on discharge to an IRF were patient age and the presence of a PM&R consultation. Therefore, this emphasizes the importance of obtaining a physiatry consultation in determining candidacy for inpatient rehabilitation, as it can give patients a higher quality of care and lead to a better recovery.

## FUNDING INFORMATION

Shirley Ryan Ability Lab Research Fund and Shirley Ryan Ability Lab Externship Program.

## DISCLOSURE

The authors declare that there is no conflict of interest.

## ETHICS STATEMENT

Our research study is not a clinical trial because we retrospectively examined patient charts, and no interventions were provided to the patients or tested. Consequently, per our institution's institutional review board, no patient consent or informed consent was needed to conduct our study. Lastly, the data of our study are available from the corresponding author, A.M.G., upon request.
